# Decoding the Role of MDSCs in Bone Metastasis: Multicellular Interactions and Clinical Implications

**DOI:** 10.3390/ph19050723

**Published:** 2026-05-02

**Authors:** Samaa Alotab, Mariam Zainab, Labibah Labib Khamies, Rasha Alissa, Khalid Said Mohammad

**Affiliations:** 1 Department of Biochemistry, College of Medicine, Alfaisal University, Riyadh 11533, Saudi Arabia; salotab01@alfaisal.edu (S.A.); mariamzainab1002@gmail.com (M.Z.); 2 Department of Clinical Skills, College of Medicine, Alfaisal University, Riyadh 11533, Saudi Arabia; lalswelhi@alfaisal.edu; 3 Department of Anatomy, College of Medicine, Alfaisal University, Riyadh 11533, Saudi Arabia; ralissa01@alfaisal.edu

**Keywords:** bone metastasis, myeloid-derived suppressor cells (MDSCs), tumor immune microenvironment (TIME), immunotherapy resistance, osteoclastogenesis and bone remodeling, myeloid immunometabolism (hypoxia–lipid axis)

## Abstract

Bone metastasis remains a major cause of morbidity in advanced cancer, driven not only by tumor–bone crosstalk but also by profound immune remodeling within the marrow. Myeloid-derived suppressor cells (MDSCs), including polymorphonuclear (PMN-MDSC) and monocytic (M-MDSC) subsets, are increasingly recognized as central effectors of this process, integrating inflammatory signals with metabolic and stromal cues to enforce immune suppression and support skeletal colonization. In this review, we synthesize current evidence that bone metastases transform the bone marrow into an “MDSC amplifier,” where vascular and endosteal niches, CXCL12-rich stromal compartments, hypoxia, and adipocyte-derived lipids collectively promote MDSC recruitment, persistence, and functional maturation. We discuss the dominant suppressive programs deployed by MDSCs in bone (e.g., arginase-1 activity, reactive oxygen/nitrogen species, and checkpoint ligand expression), and how these mechanisms converge to impair cytotoxic T-cell and NK-cell responses while fostering regulatory T-cell dominance. Importantly, because the marrow is a hematopoietic organ, bone lesions can also generate systemic consequences through myeloid spillover, providing a mechanistic basis for reduced responsiveness to immune checkpoint blockade in bone-dominant disease. We then evaluate pharmacologic strategies to target MDSCs in the context of bone metastasis, including approaches that block trafficking (e.g., CCR2/CXCR2 axes), deplete or reprogram suppressive myeloid states (e.g., STAT3-directed strategies, differentiation therapy), and disrupt bone-resorptive feedback loops (e.g., receptor activator of NF-κB ligand (RANKL) inhibition and bisphosphonates), emphasizing rational combinations and sequencing to limit marrow toxicity. Finally, we highlight emerging single-cell and spatial profiling tools that can resolve bone-specific heterogeneity in MDSCs and guide biomarker-driven, mechanism-informed therapeutic development.

## 1. Introduction

Bone metastasis develops when tumor cells disrupt the physiological remodeling process through the same molecular mechanisms used by native bone cells [1]. It is the third most frequent site of metastasis, falling behind only the lung and liver [2,3,4]. The overall incidence of bone metastasis is not definitively known. However, prostate and breast cancer are responsible for the majority of skeletal metastases, accounting for up to 70% of cases [2,3,4]. Among patients with bone metastases, the most common primary tumors arise from the lung (44.4%), prostate (19.3%), breast (12.3%), kidney (4.0%), and colon (2.2%) [5]. Additionally, bone metastases are frequently detected in patients with advanced solid tumors and most commonly involve the axial skeleton, reflecting the distribution of red bone marrow, where highly vascular tissue provides a microenvironment that promotes cellular growth [5].

Bone metastases are classified as osteolytic, osteoblastic, or mixed, based on their primary effect on normal bone remodeling [2,3,4]. Osteolytic lesions are characterized by the destruction of normal bone and are present in multiple myeloma, renal cell carcinoma, melanoma, non-small cell lung cancer, and the great majority of breast cancers [2,3,4]. Conversely, osteoblastic or sclerotic lesions are characterized by the deposition of new bone and are present in prostate cancer, carcinoid tumors, and small cell lung cancer. Mixed lesions occur if a patient has both osteolytic and osteoblastic lesions, or if an individual metastasis has components of both; while breast cancer is predominantly osteolytic, 15–20% of women present with osteoblastic or mixed lesions [2,3,4]. The clinical burden of this disease is significant, as bone metastases are a major cause of morbidity characterized by severe bone pain, impaired mobility, pathologic fractures, spinal cord compression, bone marrow aplasia, and hypercalcemia. Pathologic fractures occur in 10–30% of all cancer patients, with the femur accounting for over half of all cases.

Understanding the pathogenesis requires examining existing conceptual models, such as the “seed and soil” hypothesis, which suggests that the microenvironment provides fertile ground for metastatic cancer cells to survive and grow. Distinct anatomical features also play a role; for instance, blood vessels in the bone marrow are fenestrated and lack the usual supporting structure of capillaries, increasing the likelihood of tumor cell extravasation through the vessel wall [1].

Myeloid-derived suppressor cells (MDSCs) represent a heterogeneous population of immature myeloid cells that have emerged as pivotal regulators of metastatic progression and immune escape in cancer, particularly within the bone microenvironment [6,7]. These cells, which accumulate in bone marrow, blood, and secondary lymphoid organs in response to chronic tumor-derived inflammation, occupy a unique intersection between immune suppression and direct promotion of bone destruction. This duality positions them as contributors to osteolytic metastases [8,9]. Clinically, elevated circulating and intratumoral MDSC frequencies are consistently associated with advanced disease stage, greater metastatic burden, and poor prognosis across multiple malignancies, with bone involvement conferring particular therapeutic resistance [8,10]. In bone metastases, the immunosuppressive functions of MDSCs are compounded by their capacity to differentiate into osteoclasts (OCs), the cells primarily responsible for bone resorption, thereby enabling direct participation in the “vicious cycle” of osteolysis and tumor growth that characterizes skeletal metastatic disease [11,12]. In the bone microenvironment, MDSCs establish an immunosuppressive state, creating a “cold” immune milieu that is relatively refractory to checkpoint inhibitor therapy [13,14]. Beyond immune regulation, MDSCs promote angiogenesis, remodel the extracellular matrix, and enhance neovascularization, collectively facilitating invasion and metastatic seeding [8,10]. The bone microenvironment further confers unique protective mechanisms that sustain MDSC survival and suppressive function despite therapeutic interventions effective in soft tissue models, highlighting the necessity of bone-specific MDSC-targeting strategies [15]. Given these multifaceted roles, spanning immune suppression, osteoclastogenesis, pro-angiogenic activity, and resistance to standard therapies, MDSCs have become a central focus of combination immunotherapy strategies [9,10]. Understanding the developmental pathways, phenotypic heterogeneity, and tissue-specific crosstalk that govern MDSC biology in bone metastases is therefore essential for identifying rational therapeutic targets [6,7]. This review synthesizes the current understanding of MDSC immunobiology in bone metastasis, emphasizing subset-specific functions, stromal interactions, and emerging therapeutic approaches aimed at disrupting the pro-metastatic MDSC phenotype and restoring effective anti-tumor immunity. We argue that bone metastases convert the marrow into an MDSC-generating and MDSC-conditioning organ, where (1) vascular/endosteal niche remodeling biases myelopoiesis toward suppressive lineages, (2) bone-specific cues (hypoxia, mineralized matrix signals, osteolineage cytokines) stabilize suppressive and osteoclastogenic programs, and (3) marrow egress of ‘bone-conditioned’ MDSCs contributes to systemic resistance to immune checkpoint blockade. We therefore organize the review from (i) core MDSC suppressive mechanisms to (ii) bone niche specialization, to (iii) metabolic/stromal programming, to (iv) TIME-level crosstalk and systemic immunotherapy resistance, concluding with therapeutic and research directions.

In this review, we use the term ‘MDSC amplifier’ to describe the capacity of bone metastases not only to recruit suppressive myeloid cells but also to expand, retain, functionally condition, and systemically release them through marrow-specific niche remodeling. By ‘bone-conditioned’ MDSCs, we refer to MDSC populations exposed to the vascular, osteolineage, adipocytic, hypoxic, and mineralized cues of the bone marrow microenvironment, which may reinforce suppressive, osteoclastogenic, or differentiation-prone programs beyond those observed in primary tumors or non-skeletal metastatic sites. Direct human evidence defining these populations remains limited; therefore, the term is used here as a mechanistic framework rather than a fully standardized cell-state designation.

## 2. MDSC Biology in Cancer

MDSCs are immature myeloid cells functionally defined by their immune-suppression capabilities [16]. In mice, polymorphonuclear MDSCs (PMN-MDSCs; CD11b^+^Ly6G^+^Ly6C^low^) produce elevated ROS with minimal NO, while monocytic MDSCs (M-MDSCs; CD11b^+^Ly6G^−^Ly6C^high^) produce high NO with lower ROS [17]. Human counterparts are identified as PMN-MDSCs (HLA-DR^−^CD11b^+^CD15^+^) and M-MDSCs (HLA-DR^−^CD14^+^CD33^+^) [16]. Human MDSC phenotyping remains challenging because current marker-based definitions do not cleanly distinguish PMN-MDSCs from neutrophils or M-MDSCs from monocytes, and MDSCs can also transition toward TAM-like states or osteoclastogenic lineages in the tumor and bone microenvironments, making definitive cell assignment in human samples difficult [18,19]. For this reason, surface-marker panels alone are often insufficient, and functional suppression, developmental context, and increasingly transcriptomic or spatial profiling are needed to define MDSC states more rigorously in human disease. Critically, PMN-MDSCs require close cell-to-cell contact for ROS-dependent suppression, with a rapid half-life, whereas M-MDSCs act at a distance via NO-mediated mechanisms, with prolonged effects [16]. Both subsets express ARG1, which depletes L-arginine, an essential substrate for CD3ζ-chain synthesis and T cell proliferation [16,20]. A significant contradiction emerged during clinical translation: ARG1 inhibition achieved tumor regression in lung cancer models but failed in vitro, highlighting context-dependent efficacy [20]. Isoforms of NO synthase from M-MDSCs generate peroxynitrite (PNT), which nitrosylates T cell receptors and causes apoptosis [16,17].

ROS production via NADPH oxidase (NOX2) mediates PMN-MDSC suppression through multiple mechanisms [17]. MDSCs from NOX2-deficient mice produced substantially lower ROS and failed to suppress interferon-γ in CD8^+^ T cells [17]. Paradoxically, ROS threatens MDSC survival; MDSCs upregulate glycolysis to protect against ROS-induced apoptosis while maintaining immunosuppressive ROS production [17]. ROS scavenging forces MDSC differentiation into macrophages and dendritic cells, indicating that sustained ROS production is essential for MDSC differentiation. HIF-1α and Nrf2 drive transcriptional reprogramming, enhancing MDSC function and survival [17].

Indoleamine 2,3-dioxygenase 1 (*ido1*) catabolizes tryptophan, suppressing CD8^+^ T cells and natural killer cells while inducing regulatory T cells [21]. In lung cancer specifically, MDSC-associated IDO is essential for regulatory B cell infiltration, as demonstrated by genetic deletion of IDO in myeloid cells, which impaired Breg differentiation [22]. However, IDO inhibitors (epacadostat and navoximod) demonstrated limited single-agent efficacy (~30% stable disease) and required combination with immunotherapy, indicating IDO-dependent suppression represents one of multiple overlapping mechanisms [21].

MDSCs accumulate in distant organs before metastatic arrival, establishing pre-metastatic niches [23]. In breast cancer, bone metastasis lesions are linked to G-CSF-mobilized MDSC recruitment and lysyl oxidase (LOX)-mediated extracellular matrix remodeling [23]. The bone microenvironment’s focal hypoxia and active remodeling preferentially expand MDSCs through S100A8/A9-TLR4/RAGE signaling and HIF-1α-induced CD39L1 expression [23]. Once established, MDSCs secrete pro-metastatic factors, including TGF-β, VEGF, and MMP9 [23].

MDSC biology reflects context-dependent immunosuppressive and pro-metastatic mechanisms varying by tumor type and anatomical site [16,23]. Key contradictions include arginase inhibitor efficacy in models versus cell lines [20], limited IDO inhibitor single-agent activity [21], differential TIGIT versus PD-1 efficacy [24] and context-dependent MDSC roles requiring multi-targeted therapeutic approaches that address recruitment, differentiation, and specific mechanisms rather than single-target approaches [23].

Stimulation and core immunosuppressive mechanisms of MDSCs are broadly conserved across tumor sites, and processes described in primary tumors and other metastatic organs are therefore considered likely to operate in bone. However, extrapolation remains incomplete because the BME uniquely couples MDSC-like populations to bone remodeling, osteoclasts’ activity, osteolysis, and interactions with hematopoietic and stromal niches. Moreover, hypoxia, mineralized matrix, and bone-specific signaling are thought to further shape their phenotype, and bone-metastatic MDSCs remain insufficiently characterized. Accordingly, the central question is not whether canonical ARG1/ROS/NO/PD-L1 programs exist in bone, but how marrow niche architecture ‘locks in’ these programs and couples them to the osteoclasts’ differentiation and systemic myeloid spillover.

## 3. Bone Marrow Microenvironment as a Unique MDSC Niche

**Figure 1:**
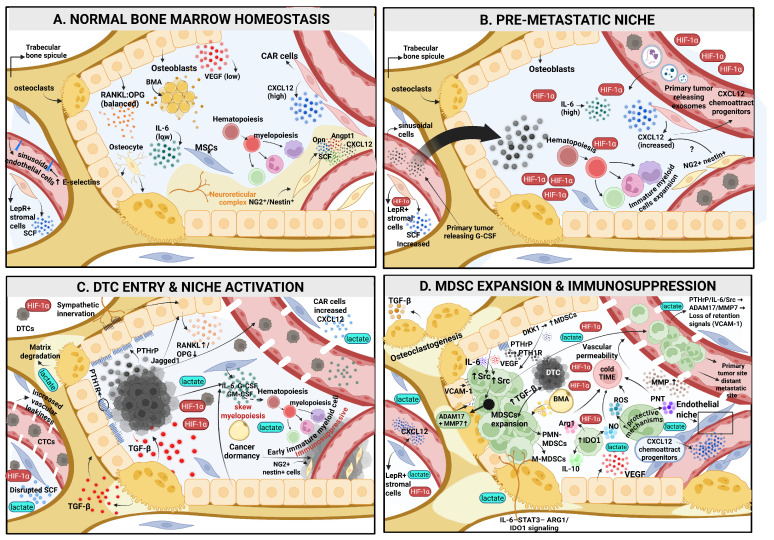
Stepwise remodeling of the bone marrow niche into an MDSC-amplifying metastatic ecosystem. (**A**) Normal bone marrow homeostasis is characterized by balanced osteoblast–osteoclast activity, CXCL12-rich stromal support from CAR and LepR+ cells, NG2+/Nestin+ neuroreticular organization, SCF/Angpt1/OPN-mediated retention signals, low IL-6 and VEGF levels, and physiologic hematopoiesis/myelopoiesis. (**B**) In the pre-metastatic niche, primary tumor-derived G-CSF and tumor exosomes are associated with increased CXCL12 and IL-6, expansion of immature myeloid cells, early stromal/niche perturbation, and a hypoxic HIF-1α-associated marrow environment that favors progenitor recruitment. (**C**) During DTC entry and niche activation, circulating tumor cells extravasate through a leakier sinusoidal endothelium; disseminated tumor cells engage osteolineage and stromal elements through PTHrP/PTH1R, Jagged1, TGF-β, and a RANKL↑/OPG↓ imbalance, while sympathetic inputs, lactate accumulation, and CXCL12-rich stromal niches promote dormancy, early colonization, and skewed myelopoiesis. (**D**) In the established metastatic niche, PMN-MDSCs and M-MDSCs expand within a hypoxic, lactate-rich marrow and deploy ARG1, NO, ROS/peroxynitrite, IDO1, IL-10, TGF-β, VEGF, and MMP-dependent programs that enhance osteoclastogenesis, vascular permeability, loss of VCAM-1-mediated retention, and formation of a cold tumor immune microenvironment. The schematic also highlights DKK1-driven MDSC expansion, PTHrP/IL-6/Src-dependent ADAM17/MMP7 upregulation, endothelial niche remodeling, and systemic spillover of marrow-conditioned myeloid cells to the primary tumor and distant metastatic sites. Created in BioRender. Mohammad, K. (2026) https://BioRender.com/nxmmcfm (accessed on 5 April 2026).

In adult bone marrow, hematopoietic stem cells (HSCs) are maintained within highly specialized microenvironments, collectively referred to as the hematopoietic niche, which is broadly organized into two principally and spatially distinct compartments: the vascular niche and the endosteal (osteolineage) niche. Both niches cooperatively regulate HSC quiescence, self-renewal, differentiation, and overall immune homeostasis, and are affected by pathological states such as metastatic cancer [25,26,27]. This ecosystem is formed by multiple interacting cell types, including osteoblasts (OBs), osteoclasts (OCs), endothelial cells (EC), mesenchymal stromal cells (MSCs), hematopoietic cells (HPC), and bone marrow adipocytes (BMA), embedded within a uniquely mineralized, calcium-rich, mechanically rigid, hypoxic, and acidic microenvironment with a distinct extracellular matrix architecture, together generating a permissive yet highly selective “soil” that shapes immune regulation and supports tumor cell seeding and metastasis [28,29]. Once disseminated tumor cells (DTCs) arrive in bone, they must adapt to these biophysical and cellular constraints, frequently entering prolonged dormancy or persisting as micrometastases before ultimately evolving into overt metastatic lesions [30]. In parallel with these niche-specific changes, MDSCs represent a central immunosuppressive component of the bone metastatic microenvironment and will be discussed as a key integrative axis throughout this review. In the sections below, a niche-centered perspective is adopted to systematically analyze the bone marrow environment (BME). The different compartments of both vascular and endosteal niches are first briefly characterized by their physiological functions, then discussed in terms of their remodeling during cancer progression, and finally linked to the regulation of MDSCs. The stepwise remodeling of the bone marrow niche from physiological homeostasis to an MDSC-rich metastatic ecosystem is summarized in Figure 1.

### 3.1. Vascular Niche

In the bone marrow, the nutrient artery branches into arterioles, which subsequently form a dense sinusoidal network that forms the core vascular architecture in which skeletal stem cells reside and interact with niche cells [25,31,32]. Under tumor-derived stimulation, this vascular microenvironment supports emergency myelopoiesis, MDSC accumulation, differentiation, functional polarization, and expansion, thereby directly linking bone marrow vascular remodeling to immunosuppression in the metastatic microenvironment [33].

Bone marrow sinusoids form a highly branched, permeable capillary network with fenestrated endothelium that facilitates the efficient exchange of cells and factors, serving as the principal site for HSC homing, engraftment, and mobilization [34,35]. Their high permeability and low shear stress create a niche that promotes HSC/HPC activation and differentiation, in part through plasma leakage, which increases local ROS production [36,37]. This vascular niche is regulated by ECs, with sinusoidal endothelial cells (SECs) actively producing CXCL12 and E-selectin, as well as Jagged-1 and transcription factors such as Klf6 [27,38,39]. This SEC layer is not supported by pericytes, which facilitates two-way cell trafficking and promotes circulating tumor cells (CTCs) invasion, extravasation, and docking within the bone marrow, initiating bone metastasis [40]. Moreover, this vascular leakiness will be enhanced by the effect of tumor cells on other cells, including IL-6, TNF-α, and OCs-derived inflammatory cytokines [41,42]. Importantly, MDSCs release IL-6 at the primary tumor site and, if similarly released in the pre- or metastatic bone marrow niche, are likely to recapitulate the previously described cytokine-driven effects on SECs. They also enhance angiogenesis and vascular leakiness via VEGF, MMPs, and direct disruption of endothelial junctions [43,44].

The stromal component of this niche is principally composed of multipotent MSCs, which can generate OB, BMA, and chondrocytes, although their primary function within the niche is to provide critical regulatory support [26,32,35,45]. The heterogeneous perisinusoidal MSCs population in the perisinusoidal niche includes leptin receptor and (LEPROT)–PDGFRα-expressing cells (LepR^+^), which are a main source of stem cell factor (SCF) (KIT-ligand), (CXCL12) (SDF-1)-abundant reticular (CAR) cells, which are the main source of CXCL12, CD146^+^ pericytes, and Nestin-low stromal cells [26,39,45,46,47]. Functionally, they use CXCL12 and SCF to regulate HSC retention and quiescence [35,46,47,48]. This physiological CXCR4/CXCL12 homing axis, along with matrix molecules such as integrin family members and adhesion molecules such as E-selectin, is co-opted during bone metastasis, as CTCs are drawn to the high perivascular CXCL12 gradient to establish metastatic footholds [40]. Tumor-derived cytokines reprogram MSCs toward a myeloid-biased state, promoting MDSC expansion, immunosuppression, and metastatic progression, a process further amplified by inflammatory mediators such as IL-1β, IL-6, IL-10, SCF, and CXCL12, which enhance vascular permeability and niche inflammation [45,49,50]. Thus, perisinusoidal MSCs, including LepR^+^ and CAR populations, are thought to support MDSC expansion and accumulation in tumors by producing these chemotactic signals. These findings suggest that marrow MSC subsets play distinct rather than uniform roles in metastasis. LepR^+^ and CAR-like stromal cells are more linked to HSC retention and myeloid trafficking, whereas NG2^+^/Nestin^+^ populations are more associated with quiescence, dormancy signaling, and stromal regulation of MDSC-supportive states.

In the normal physiological state, a distinct MSC subtype resides in the periarteriolar BM niche, closely associated with neuronal fibers and expresses markers such as neural/glial antigen 2 (NG2) and nestin [45]. These NG2+ nestin+ perivascular cells are essential organizers of a critical niche that maintains HSC quiescence by secreting SCF, CXCL12, Angiopoietin-1 (Angpt1), VCAM1, and Osteopontin (Opn), among others. Their depletion, or the specific deletion of SCF from them, alters HSC localization away from arterioles, reducing the functional HSC pool in the BM. This specific, dormancy-promoting microenvironment is closely associated with the pathophysiology of cancer metastasis to bone, where it fosters an immunosuppressive landscape [27,51]. Further, they can regulate tumor cell behavior by secreting TGF-β2 and BMP7, which signal via TGFBRIII and BMPRII to activate SMAD, p38, and p27 pathways and promote cancer cell dormancy. In contrast, in other contexts, NG2^+^ stromal cells interact with DTCs, thereby enhancing their proliferation and migration [52,53]. As discussed above, NG2^+^/Nestin^+^ cells are also local sources of SCF and CXCL12, suggesting that this niche could influence MDSC expansion and recruitment through chemokine and growth-factor axes known to regulate MDSC development and trafficking; however, direct evidence linking specific NG2^+^ stromal populations to MDSC regulation in bone metastasis remains limited, and this crosstalk is poorly defined.

### 3.2. Endosteal Niche and Osteolineage Surface

OB and osteolineage cells may support HSCs by expressing various regulatory molecules, including TPO, Angpt1, OPN, CXCL12, and SCF, which regulate stem cell pool size, although this role remains uncertain [27,35,39]. In cancer, a “vicious cycle” forms between tumor cells, OB, and OC, in which tumor signals, such as SCUBE2, contribute to osteoblastic lesions and protective niches that shield cancer cells from immune surveillance, as seen in prostate cancer [25,54]. Conversely, in osteolytic metastases, such as those from breast cancer, tumor-derived ligands, such as Jagged1, activate Notch signaling in OB, enhancing the production of pro-osteoclastogenic factors (e.g., RANKL, IL-6) and accelerating bone destruction [55]. OB also regulates tumor cell fate by secreting factors such as GAS6 and TGFβ2: GAS6 binds to the tyrosine kinase receptor Axl expressed on DTCs, while TGFβ2 activates signaling pathways, including the TGFβRIII-p38 MAPK axis, inducing and maintaining dormancy [56,57]. Beyond regulating bone remodeling and tumor dormancy, osteoblasts actively shape the metastatic niche through bidirectional interactions with MDSCs. It is important to distinguish systemic MDSC mobilization from local niche regulation: tumor-derived cytokines can mobilize MDSCs from the bone marrow to distant pre-metastatic sites, and tumor-derived PTHrP activates PTH1R in bone marrow stromal cells and osteoblasts, inducing VEGF-A and IL-6 production and promoting Src phosphorylation in monocytic MDSCs, which upregulate ADAM-17 and MMP7, disrupt VCAM-1–mediated tethering, and facilitate mobilization, a process distinct from their regulation within established bone metastases [56]. In contrast, within the bone metastatic niche, osteoblast-derived factors such as DKK1 enhance MDSC recruitment, expansion, and immunosuppressive activity, and inhibition of DKK1 disrupts this pathway, reducing MDSC-mediated immunosuppression and tumor progression [25,58]. Together, this reciprocal crosstalk establishes a feed-forward loop in which osteoblast-mediated niche remodeling and MDSC-driven immunosuppression and osteolysis synergize to promote metastatic progression (Figure 1C,D).

Embedded within the mineralized bone matrix, osteocytes function as master regulators, sensing mechanical and hormonal stimuli to coordinate bone remodeling and granulopoiesis by expressing critical factors such as RANKL, M-CSF, IL-19, and by producing cytokines that support osteoclastogenesis, neutrophil development, and the regulation of phosphate and calcium balance [4,25,59,60]. Osteocytes may influence tumor progression indirectly by regulating OB and OC activities, thereby promoting bone resorption and the release of matrix-derived growth factors that support tumor progression. Osteocyte-derived factors, including sclerostin and DKK-1, as well as dysregulation of Notch and Wnt signaling pathways, have been implicated in bone disease and tumor progression, although their precise in vivo roles remain incompletely defined. Evidence directly linking osteocytes to MDSC regulation in bone metastasis remains sparse; however, under stress conditions, osteocytes are known to stimulate the release of mediators such as RANKL, IL-6, M-CSF, and TGF-β, which are recognized drivers of MDSC accumulation, trafficking, and immunoregulatory activity. This suggests that osteocyte-derived signals can indirectly influence MDSC dynamics in the bone metastatic environment [61,62,63].

In physiological bone remodeling, OC, derived from hematopoietic precursors via M-CSF signaling through c-Fms, and RANK, which induces the expression of osteoclastogenic transcriptional factors, are essential for bone resorption and contribute to the HSC niche [60,64]. MDSCs are integral components of this process and closely linked to osteoclast biology. Upon tumor cell infiltration, MDSCs and monocyte–macrophage lineage cells can differentiate into functional OC, thereby contributing to osteolytic lesion expansion, inflammation, vascular permeability, and DTC extravasation [41,65]. In addition, MDSCs reinforce the vicious cycle by producing elevated levels of TGF-β, which upregulates PTHrP and Gli2 expression in tumor cells, further driving osteoclastogenesis and bone resorption [25]. MDSCs also promote tumor progression by inducing EMT, enhancing invasion, and conferring resistance to apoptosis [60]. Their accumulation has been observed in bone metastases of breast cancer, prostate cancer, and multiple myeloma, and tumor-derived soluble factors such as G-CSF, VEGF, IL-6, and TNFα contribute to their recruitment and expansion, particularly in tumors with high osteotropic potential [25]. From a therapeutic perspective, beyond reducing osteoclast activity, RANKL-targeted therapies may also influence the immunological tumor microenvironment, including pathways involved in MDSC-mediated immunosuppression and osteolysis discussed above, although their precise immunomodulatory effects remain insufficiently defined and warrant further investigation. Similarly, amino-bisphosphonates have been shown to reduce MDSC expansion, although high MDSC accumulation may limit their therapeutic effectiveness [23,41].

### 3.3. Adipocytic, Megakaryocytic, and Neural Stromal Networks

BMAs represent a distinct adipocyte population with unique metabolic and paracrine features compared to peripheral white adipocytes [66]. They secrete a broad spectrum of adipokines (leptin, adiponectin), cytokines (IL-6, IL-1β, TNF-α, RANKL), chemokines (CXCL1, CXCL2, CXCL5, CXCL12, CX3CL1, CCL2, CCL7), growth factors (IGF-1, FGF-2), and novel adipokines including ANGPTL2/4, chemerin, FABP4, LCN2, resistin, and visfatin, through which they regulate both osteoblastogenesis and osteoclastogenesis [28,42,50,66]. These factors mediate MDSC recruitment to the bone marrow and promote TAM polarization, angiogenesis, and tumor progression [42,50,63]. These effects may be reinforced by cooperation with MDSC-derived MMP-9 and by BMA-fueled lipid–ROS programs that sustain MDSC immunosuppression [63,67].

Peripheral nerves extensively innervate the skeleton, and neural inputs integrate bone remodeling, hematopoiesis, and immune homeostasis [26]. Sympathetic nerves and Nestin^+^ perivascular stromal cells form the neuroreticular complex, a central component of the HSC niche, where quiescent HSCs localize near arterioles and are regulated by circadian noradrenaline signaling through β3-adrenergic receptors on Nestinhigh/NG2^+^ MSCs, driving rhythmic CXCL12 expression and HSC egress, while β2-adrenergic receptors inhibit osteoblast formation [27,35]. Non-myelinating SCs maintain HSC quiescence through TGF-β/SMAD signaling, while nociceptive nerves cooperate with sympathetic fibers to drive G-CSF-induced HSC mobilization [35,68]. Parasympathetic inputs also contribute to promoting glucocorticoid-dependent HSC mobilization [39]. In cancer, chronic stress activates sympathetic signaling, stimulating β2AR on OB cells to increase RANKL and osteoclastogenesis [29]. It also stimulates angiogenesis, metabolic reprogramming, IL-6 secretion, infiltration of Tregs and MDSCs, suppression of CD8^+^ T cells and NK cells, macrophage and neutrophil polarization, chemoresistance, and tumor stemness [69]. MDSCs accumulate in the pre-metastatic niche to promote neovascularization and are directly expanded and activated by sympathetic β-adrenergic signaling through IL-6–STAT3 ARG1/IDO1 signaling [69]. Regarding SCs, they critically regulate this axis by upregulating TNF-α, CXCL12, CCL2, CCL3, CCL4, CXCL2, SDF-1, CXCL13, and MAG to recruit MDSCs and boost their ability to inhibit T-cell proliferation [70,71].

MKs are large, rare hematopoietic cells in the BM, located around the perisinusoidal regions, and constitute a key regulatory component of the HSC niche, where they interact with other cells and structures to preserve stem cell quiescence [26]. In cancer, reprogrammed MKs release pro-fibrotic and pro-inflammatory mediators (IL-1, IL-6, S100A8/A9, thrombospondin-1) that shape myeloid differentiation, angiogenesis, and tumor behavior, forming a context-dependent pro- and anti-metastatic MK–platelet axis [72]. Meanwhile, TPO inhibits OCs, promotes OBs proliferation, and drives the release of mesenchymal growth factors, as well as both pro angiogenic (VEGF, PDGF, HGF) and anti-angiogenic (TSP 1, plasminogen activator inhibitors) factors [72,73]. Although direct evidence that MKs instruct MDSCs differentiation remains limited, several converging mechanisms support a functional MK–MDSC axis in cancer. Emergency megakaryopoiesis generates CXCR4^high^ MKs that secrete IL-6 and TNF-α and physically interact with myeloid cells, providing inflammatory cues that favor myeloid expansion and immunosuppressive programming [26]. At the primary tumor site, platelets can induce bone marrow-derived cells (BMDCs) recruitment and promote tumor angiogenesis and growth through secretion of a-granules, and, to a certain extent, they can also promote the recruitment of MDSCs [74]. Evidence on the role of megakaryocytes in cancer remains limited and sometimes conflicting, and their interactions with the bone marrow in the metastatic microenvironment are even less defined, with no clear conclusions established to date [73].

Collectively, these vascular, osteolineage, adipocytic, neural, and megakaryocytic circuits do more than recruit myeloid cells; they reshape the nutrient, oxygen, lipid, and cytokine landscape that determines whether recruited myeloid precursors differentiate, die, or persist as suppressive MDSCs. This motivates a focused discussion of metabolic and stromal reprogramming as the proximate ‘execution layer’ of MDSC function in bone.

## 4. Metabolic and Stromal Reprogramming of MDSCs in the Bone Marrow Niche

Whereas Section 3 addressed the structural and cellular organization of the bone marrow niche, this section focuses on the signals by which that niche conditions MDSCs. The emphasis is on the hypoxic, cytokine-rich, metabolically altered, and stromally reprogrammed environment that promotes MDSC persistence, suppressive function, and lineage plasticity in bone metastasis.

Many of the mechanisms regulating MDSC recruitment, plasticity, and function have been introduced in the preceding sections; here, these processes are briefly integrated in the context of metabolic and stromal reprogramming within the metastatic niche. Some of these pathways have been characterized in metastatic sites other than the bone. Although similar cues exist in bone, their precise roles in the bone metastatic microenvironment remain to be fully defined.

Metabolic cues are key determinants of MDSC plasticity, as metabolic reprogramming regulates nutrient and oxygen availability and receptor signaling, thereby controlling their differentiation, survival, and immunosuppressive function [75]. Accordingly, MDSC recruitment and functional programming are regulated by a broad network of chemokines, cytokines, and stromal signals, including CXCL1–CXCR2, CXCL12–CXCR4, CCL2–CCR2, IL-6, G-CSF, POSTN, hypoxia-induced HIF-1α, LOX, and platelet COX-1/TXA2 signaling, which collectively expand MDSCs and shape their suppressive phenotype [76]. Once established in the tumor microenvironment, MDSCs mediate immunosuppression via ARG1, iNOS, ROS, PD-L1, and TGF-β/IL-10; promote angiogenesis and vascular permeability through VEGF, FGF2, and MMPs; drive inflammation through IL-1β, S100A8/A9, and SAA; and facilitate tumor cell arrest, survival, and pre-metastatic niche formation [60,76]. Although these mechanisms have been described at the tumor site, they are highly relevant to bone metastasis, as MDSCs recruited to or generated within the bone marrow niche are exposed to similar tumor-derived signals that shape their differentiation, immunosuppressive activity, and contribution to metastatic progression, augmenting the effects of OC and BMA that have been previously described. This context-dependent behavior reflects the high plasticity of MDSCs, which is reprogrammed by local stromal, metabolic, and hypoxic signals within metastatic niches. Local signals such as hypoxia, STAT3 signaling, CSF1, CSF2, and VEGF regulate their differentiation, frequently driving conversion of M-MDSCs into TAM, while blockade of the CSF1/CSF1R axis reduces MDSC and TAM accumulation and impairs their maturation. The differentiation into DCs is less frequent and is associated with diminished antigen-presenting capacity [77,78,79]. Finally, systemic factors can modulate these processes in a stage-dependent manner, as G-CSF suppresses cancer cell homing to bone when administered before tumor seeding but accelerates metastatic progression after colonization, at least partly through MDSC-mediated mechanisms [80].

In bone and other metastatic sites, metabolic and stromal reprogramming further reinforce this immunosuppressive program (Figure 1D). The hypoxic bone marrow niche, in which MSCs act as immunometabolic regulators, activates HIF signaling, glycolytic metabolism, and VEGF-A and CXCL12 production, thereby promoting MDSC and M1-to-M2 macrophage polarization and suppressive function through JNK signaling and the induction of mediators such as ARG1, Nos2, HIf1α, and IL-10 [38,39,60,61,81]. Furthermore, in an inflammatory setting, MSCs promote MDSC expansion and immunosuppressive activity via paracrine mediators, including HGF and COX-2/PGE2 [82]. Tumor-derived metabolites, including lactate, acetic acid, and lipids, amplify this process by stabilizing HIF-1α, activating PPAR-γ in FFAR2-expressing MDSCs, enhancing ARG1 expression, and reshaping immune metabolism, while inhibition of these pathways restores T-cell infiltration and reduces immunosuppression [83,84,85,86]. In parallel, stromal and tumor-derived factors, such as hyaluronan, CD44 signaling, and CXCL12-dependent HIF programs in osteolineage cells, contribute to metastatic colonization and adhesion within bone [29,86,87,88]. As a side note, tumor-derived lactate and the resulting acidification can further shape the bone metastatic niche by activating osteoclasts, impairing T-cell recognition and function independent of MDSCs and stimulating sensory neurons that contribute to tumor-associated bone pain [86].

These metabolic programs are not merely cell-intrinsic: hypoxia, lactate, and lipid uptake shape how MDSCs engage surrounding immune populations, biasing the bone metastatic tumor immune microenvironment toward T-cell dysfunction, Treg dominance, and impaired NK activity. We therefore next map the cell–cell suppressive circuitry through which MDSCs shape the bone-metastatic immune microenvironment.

## 5. Crosstalk Between MDSCs and Other Immune Cells in the Bone Metastatic TIME

The accumulation of MDSCs has a marked impact on T-cell unresponsiveness to circulating antigens, which plays a vital role in early tumor progression [89]. Several mechanisms have been implicated in MDSC-mediated suppression of CD8^+^ and CD4^+^ T-cell functions. The increased activity of ARG1 depletes L-Arginine, a key modulator of T cell proliferation, thereby effectively inhibiting T cell responses [89,90,91]. ROS, in particular, PNT generated from the reaction between NO and superoxide, induce oxidative and nitrosative stress, which then inhibits ζ-chain expression in T cells and antigen-induced cell proliferation [92]. Other factors involved in MDSC-mediated immune suppression include the upregulation of PD-L1, an activator of T cell co-inhibitory pathways that suppresses T cell activation, thereby promoting tumor cell immune escape and progression [93]. MDSCs and Tregs form a tightly interconnected suppressive axis in the tumor microenvironment. MDSCs promote Treg expansion and activation, leading to the conversion of conventional CD4^+^ T cells into FoxP3^+^ Tregs and the recruitment of preexisting Tregs to tumor sites [94]. Tregs eventually suppress T cell-mediated immune responses by secreting immunosuppressive cytokines, such as TGF-β, IL-10, and IL-35, and by inhibiting effector T cell function [95]. In turn, Tregs reinforce MDSC suppressive function by secreting IL-10 and TGFβ and by shaping myeloid cells to upregulate inhibitory B7 family molecules (including B7H1/PDL1) on MDSCs, creating a positive feedback loop that amplifies immunosuppression [94,96]. In the presence of bone metastasis, these induced FoxP3^+^ Tregs foster osteoclast differentiation via CTLA-4-dependent cell-to-cell interactions and RANKL secretion, while residing within an MDSC-rich immunosuppressive bone niche that further silences effector T cells and eventually enhances osteoclastogenesis [14].

In contrast, there is a significant knowledge gap that exists regarding MDSC interplay with NK and B cells, particularly in bone metastases, and the available data from other cancer sites indicate that MDSCs can dampen NK cytotoxicity and promote regulatory PDL1^+^ B cell phenotypes [97,98,99,100]. Still, dedicated skeletal studies remain scarce. In bone metastases, PMN-MDSCs and M-MDSCs phenotypically resemble neutrophils and classical monocytes, respectively, but are skewed toward a stably immunosuppressive state [101]. Consistent with the differentiation pathways discussed earlier, single-cell analyses have shown that M-MDSCs can give rise to TAMs and osteoclast-like cells within the bone marrow niche, further reinforcing the contribution of early MDSC expansion to the TAM- and osteoclast-rich architecture observed in established bone lesions [101,102,103]. Collectively, these data support an integrative view of the bone metastatic TIME as a highly MDSC-structured network; more myeloid-heavy and immunosuppressive than many visceral sites, underscoring the rationale for combining MDSC-targeted strategies with checkpoint blockades or other immunotherapies in skeletal metastases.

**Figure 2:**
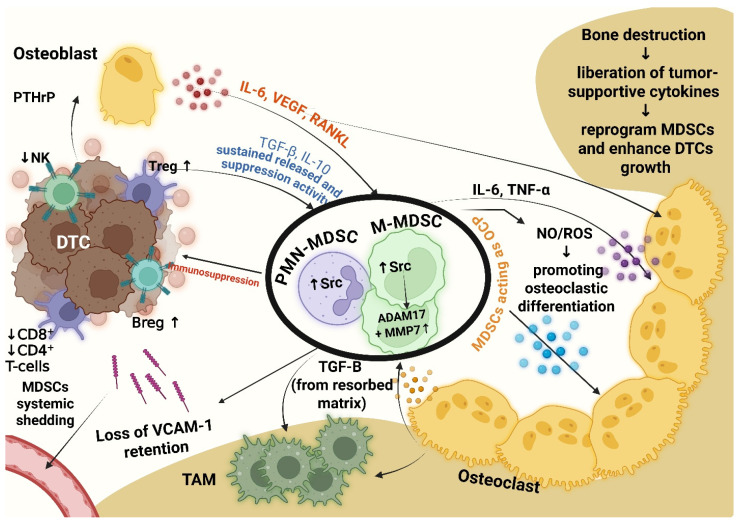
MDSC-centered multicellular circuitry driving immune suppression, osteoclastogenesis, and systemic spillover in bone metastasis. Osteoblast-associated PTHrP signaling and osteoblast-derived IL-6, VEGF, and RANKL promote activation and functional conditioning of MDSCs within the bone metastatic niche. PMN-MDSCs and M-MDSCs are shown as complementary suppressive subsets, with enhanced Src signaling and, in M-MDSCs, ADAM17/MMP7 upregulation that contributes to loss of VCAM-1-mediated marrow retention. MDSCs suppress anti-tumor immunity by reducing CD4+ and CD8+ T-cell and NK-cell activity while promoting Treg and Breg expansion. In parallel, MDSCs can differentiate into TAMs and act as osteoclast progenitor-like cells, with NO/ROS-driven osteoclastic differentiation enhancing bone resorption. Osteoclast-mediated matrix degradation releases TGF-β and other tumor-supportive factors that further reprogram MDSCs and promote DTC growth, thereby reinforcing a feed-forward cycle of immune suppression, osteolysis, and metastatic progression. The figure also highlights systemic shedding of MDSCs from the marrow, linking local skeletal immune remodeling to broader systemic immune dysfunction. Created in BioRender. Mohammad, K. (2026) https://BioRender.com/704ejst (accessed on 5 April 2026).

Importantly, this marrow-centered suppressive network is positioned to influence immunity beyond bone lesion, because bone marrow is both a metastatic site and a hematopoietic organ. This provides a mechanistic basis to consider bone metastases as a systemic driver of immunotherapy resistance. The reciprocal immunosuppressive and osteoclastogenic circuitry linking osteoblasts, MDSCs, immune cells, TAMs, and osteoclasts is summarized in Figure 2.

## 6. Systemic Effects: Bone Metastases as an MDSC-Driven Barrier to Immunotherapy

It appears that bone lesions function as a systemic, MDSC-rich barrier to effective immunotherapy, as patients with bone metastases consistently have poorer outcomes with immune checkpoint inhibitors (ICIs) across a variety of solid tumor types [104]. In comparison to patients without skeletal involvement, cohort studies in metastatic non-small cell lung cancer have shown that individuals with bone involvement had lower response rates, significantly shorter progression-free survival (PFS), and decreased overall survival (OS) to PD-1/PD-L1 inhibitors [105,106]. This clinical pattern aligns with experimental data showing that bone metastases drive a systemic reprogramming of myelopoiesis, which is characterized by expansion of circulating and marrow MDSCs, heightened myeloid skewing, and systemic T-cell dysfunction. These changes together create an MDSC-dominated immune landscape that blunts the ability of ICIs to reinvigorate T-cells at distant sites [11,102]. Studies in mouse models point in this direction: when bone metastases are present, ICIs tend to be less effective against tumors elsewhere in the body; however, when signals from the bone microenvironment that suppress immune activity are disrupted, the systemic response to ICIs is partially restored [107]. Taken together, these findings suggest that bone metastases may act as a barrier, shaped by MDSCs, that limits the effectiveness of immunotherapy.

Bone metastases can serve as a continuous source of bone-derived MDSCs, which are gradually released from the marrow into the circulation and can weaken T-cell responses beyond the local tumor microenvironment (Figure 2). This sustained release appears to be supported by signals in the bone microenvironment, including RANKL, TGF-β, and tumor-derived PTHrP, which shift myeloid development toward suppressive lineages and allow MDSCs to also behave as osteoclast precursors within the metastatic niche, as discussed previously [11,12,56]. From this perspective, the bone metastatic niche functions not simply as a passive site of tumor residence, but more like an “immunosuppressive amplifier” that keeps generating and releasing bone-conditioned MDSCs into the circulation, gradually shaping systemic immunity, a view that has not been clearly articulated in prior MDSC or bone metastasis studies.

## 7. Therapeutic Targeting of MDSCs in Bone Metastatic Disease

**Figure 3:**
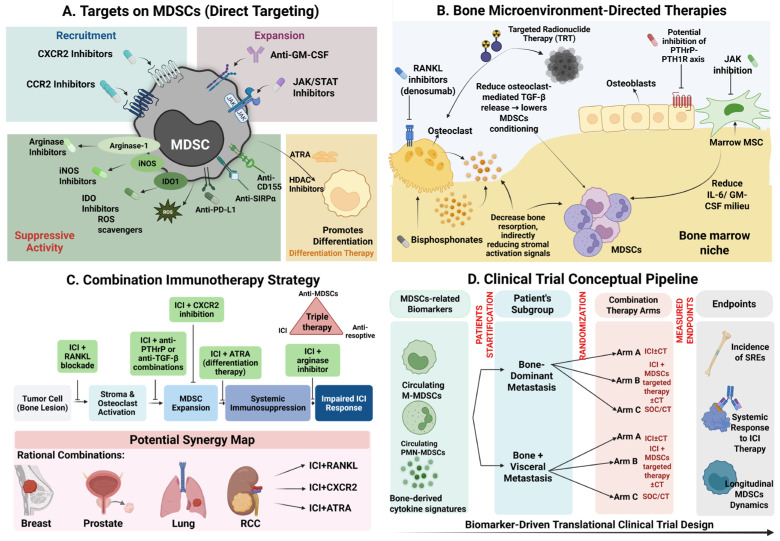
Therapeutic targeting of MDSCs in bone metastatic disease: direct interventions, niche-directed strategies, rational combinations, and a translational trial framework. (**A**) Direct MDSC-targeting approaches include blockade of recruitment (CXCR2 and CCR2 inhibitors), inhibition of expansion (anti-GM-CSF and JAK/STAT inhibitors), suppression of effector programs (arginase-1, iNOS, and IDO inhibitors; ROS scavengers; anti-PD-L1; anti-CD155; anti-SIRPα), and differentiation therapy (ATRA and HDAC inhibitors). (**B**) Bone microenvironment-directed therapies include bisphosphonates, RANKL inhibition, targeted radionuclide therapy, potential disruption of the PTHrP-PTH1R axis, and JAK inhibition to reduce IL-6/GM-CSF-rich stromal conditioning and osteoclast-mediated TGF-β release. (**C**) Rational immunotherapy combinations pair immune checkpoint inhibition with anti-MDSC or anti-resorptive strategies, including ICI + CXCR2 inhibition, ICI + arginase inhibition, ICI + RANKL blockade, ICI + ATRA, ICI + anti-PTHrP/anti-TGF-β combinations, and triple-therapy concepts integrating ICI, anti-MDSC, and anti-resorptive agents. (**D**) A biomarker-driven translational clinical trial schema is proposed in which patients are stratified by metastatic pattern (bone-dominant versus bone plus visceral disease), assessed using MDSC-related biomarkers (circulating M-MDSCs, circulating PMN-MDSCs, and bone-derived cytokine signatures), randomized across therapeutic arms, and evaluated using endpoints such as skeletal-related events, systemic response to ICI therapy, and longitudinal MDSC dynamics. Created in BioRender. Mohammad, K. (2026) https://BioRender.com/sb5537z (accessed on 5 April 2026).

Therapeutic strategies targeting MDSCs in bone metastasis are summarized in Figure 3.

### 7.1. Direct MDSC-Targeting Approaches

Lower doses of certain chemotherapy drugs appear to directly reduce MDSCs without harming active lymphocytes [9]. Gemcitabine and 5-FU have been shown to be effective in preclinical models at specific doses, reducing splenic and tumor-infiltrating MDSCs, improving CD8^+^ T-cell function, and enhancing responses to immunotherapy [108,109]. Similar MDSC-lowering effects have been reported with cyclophosphamide, paclitaxel, docetaxel, and even platinum-based drugs, suggesting that these drugs can serve not only as cytotoxic agents but also as components of MDSC-modulating regimens that may be particularly relevant in the MDSC-rich setting of bone metastatic disease [9,110]. Furthermore, all-trans retinoic acid (ATRA) drives MDSC differentiation toward less immunosuppressive dendritic cells or macrophages by activating ERK1/2 signaling and reducing ROS levels. Early clinical studies involving metastatic melanoma have shown reduced circulating MDSC levels, though bone metastasis-specific studies remain scarce [111]. Another key approach involves inhibitors of CXCR2, CCR2, and CSF1R, which can block MDSC migration into tumors and bone marrow by disrupting chemokine gradients and survival signals that attract immature myeloid cells to metastatic sites [101,112,113]. These pathways are particularly relevant in bone metastases, where CCR2^+^ M-MDSCs and CXCR2^+^ PMN-MDSCs accumulate in response to tumor and stroma-derived CXCL12, CCL2, and CSF1 growth factors. In parallel, small-molecule inhibitors of ARG1 and iNOS, together with ROS scavengers, counteract MDSC-mediated immunosuppression by restoring L-arginine availability to T-cells and reducing the oxidative or nitrosative damage to TCR signaling, while checkpoint-oriented strategies targeting the TIGIT–CD155 and CD47–SIRPα axes on MDSCs further block contact-dependent suppression and potentially enhance macrophage phagocytosis of tumor cells within the bone niche [114,115].

Immune checkpoint inhibitors (ICIs) have arisen as a central element of modern anticancer immunotherapy, with several agents acting on the PD-1/PD-L1 and CTLA-4 axes now approved across multiple solid tumors [116]. Anti-PD-1 agents such as nivolumab, pembrolizumab, cemiplimab, and dostarlimab function by blocking PD-1 signaling on activated T cells, thereby restoring effector function and reversing T-cell exhaustion within the tumor microenvironment [116]. Similarly, anti-PD-L1 agents, including atezolizumab, durvalumab, and avelumab, inhibit ligand-mediated suppression of T-cell activity while potentially preserving PD-L2 signaling, which may influence toxicity profiles [116]. In parallel, CTLA-4 blockade with agents such as ipilimumab and tremelimumab enhances early T-cell priming and expansion in lymphoid tissues, complementing the peripheral reinvigoration achieved by PD-1/PD-L1 inhibitors [116]. These agents are widely used either as monotherapy or in combination regimens and are being further expanded through ongoing clinical trials and next-generation checkpoint-targeting strategies. Notably, numerous next-generation checkpoint-targeting compounds and combination strategies are currently under clinical development, including bispecific antibodies and inhibitors of additional immune checkpoints such as LAG-3, TIM-3, and TIGIT, reflecting continued efforts to expand and refine checkpoint blockade in immunotherapy [117]. Building on these advances, ICIs are of particular interest in the context of bone metastasis, where their activity may be constrained by the immunosuppressive marrow niche [118]. Consequently, to address these limitations, combinations integrating ICIs with MDSC-targeting approaches are to be studied to enhance antitumor immunity and improve responses in bone-dominant disease.

### 7.2. Bone-Targeted Therapies That Indirectly Impact MDSCs

Among bone-targeted therapies, bisphosphonates and RANKL inhibitors offer dual benefits by inhibiting osteoclast-mediated bone resorption and by emerging immunomodulatory effects on MDSCs [11,119]. Bisphosphonates such as zoledronic acid block farnesyl pyrophosphate synthase in the mevalonate pathway, thereby disrupting protein prenylation in myeloid-lineage cells and reducing MDSC survival and expansion in the bone marrow, while also limiting tumor-driven recruitment by decreasing osteoclast-mediated bone resorption and related signaling [119,120,121]. This combined effect may help slow tumor progression, especially when used with chemotherapeutic agents such as gemcitabine [119]. Denosumab, a monoclonal antibody targeting RANKL to inhibit osteoclast differentiation, delays skeletal-related pathology in patients with bone metastases. However, its direct effects on MDSCs remain underexplored to date [122]. Moreover, some radiopharmaceutical therapy agents (TRT), such as radium-223 (Ra-223) dichloride and lutetium-177 (Lu-177), can target bone metastases with alpha- or beta-particle radiation, inducing DNA damage in tumor cells and nearby osteoclasts to alleviate skeletal complications [123,124,125]. These agents have the potential to deplete MDSC pools in the bone marrow niche by inducing myeloid cell apoptosis and altering hematopoiesis, thereby alleviating local immunosuppression [124,125].

Combining ICIs with anti-MDSC agents addresses the bone niche’s profound immunosuppression by restoring TCR signaling and enhancing the efficacy of PD-1/PD-L1 blockade, as evidenced by preclinical prostate cancer models showing reduced tumor burden [112,123]. Adding a third component, such as anti-resorptive drugs alongside ICIs and direct MDSC inhibitors, may further reshape the metastatic environment by limiting osteoclast activity and reducing MDSC levels. However, early clinical results suggest benefits in PFS while also highlighting the need for careful timing to avoid blood-related side effects [104,126,127]. Some preclinical studies suggest that combining different immune approaches may be especially useful in bone metastasis. For example, in breast cancer models with bone involvement, blocking TIGIT together with inhibiting MDSCs has shown a strong combined effect, stronger than either strategy alone [128]. At the same time, there are clear challenges. Treatments that affect myeloid cells can be toxic to the bone marrow, raising concerns about infections and unintended effects on normal blood cell production. Because of this, it is still unclear which MDSC-targeting drugs are the most realistic options in bone metastasis, or how best to combine them with existing bone-targeted therapies and ICIs. Right now, there is no clear synthesis that brings these pieces together. From a translational standpoint, these regimens could be tested in biomarker-guided trials stratified by metastatic pattern and longitudinal MDSC dynamics (Figure 3D).

Following the discussion of therapeutic strategies targeting MDSC-related pathways, it is important to revisit the experimental evidence supporting their role in bone metastasis. As discussed earlier, MDSCs promote tumor progression through multiple immunosuppressive and cytokine-mediated mechanisms. The following sections summarize preclinical and clinical studies demonstrating MDSC accumulation and functional involvement in the bone metastatic microenvironment, and further highlight how these processes manifest in a tumor- and lesion-specific manner.

A practical limitation of MDSC-directed therapy in bone metastasis is that the same marrow compartment that sustains suppressive myelopoiesis also supports normal hematopoiesis. Interventions that deplete or reprogram myeloid populations therefore risk cytopenias, infection, impaired tissue repair, or unintended disruption of host myeloid function, which helps explain why clinical translation has lagged behind mechanistic promise.

## 8. Evidence for MDSC Accumulation and Function in Bone Metastases

### 8.1. Preclinical Models

Murine models have been the basis of studying bone metastasis in breast cancer. Sawant et al. assessed MDSC behavior in breast cancer using an immunocompetent mouse model of bone metastasis [119]. In their study, Sawant et al. injected 4T1 (fLuc) in BALB/c mice, allowing the cells to spread and form bone metastases in a genetically matched mouse model. Results indicated that bone metastatic lesions and adjacent marrow exhibit marked accumulation of CD11b+ and Gr-1+ MDSCs [119]. After testing MDSCs from different parts, it was established that only MDSCs taken from the bone marrow of mice with bone metastases differentiated into osteoclasts, indicating that the bone environment is essential for MDSCs to differentiate into osteoclasts [119].

In another study by Lee et al., which assessed how BM stromal cell activation by PTH1R contributes to MDSC mobilization, it was established that tumor-derived PTHrP is critical for mobilizing M-MDSCs from the bone marrow into the circulation [56]. According to Lee et al., the activation of PTH1R triggers mobilization rather than local expansion within the bone marrow [56]. Evidence shows that M-MDSCs bind to osteoblasts through α4β1 integrin, and the activation of PTH1R on osteoblasts leads to the upregulation of proteases like ADAM17 and MMP7 in M-MDSCs, thereby disrupting M-MDSC–osteoblast adhesion. Lee et al. demonstrated that this molecular mechanism, which disrupts M-MDSC–osteoblast adhesion, illustrates how the bone marrow microenvironment regulates the supply of M-MDSCs to the tumor milieu [56].

Elsewhere, murine prostate cancer models have also revealed the existence of MDSC accumulation in bone metastasis. Sawant and Ponnazhagan synthesized evidence on the role of MDSCs in bone metastasis across different cancers, including breast, lung, and prostate [11]. They describe that in all these cancers, MDSCs suppress T-cell activity, promote tumor growth, and act as osteoclast progenitors (OCPs) [11]. According to Sawant and Ponnazhagan, HIF-1α enhances nitric oxide production, creating a feedback loop that accelerates MDSC differentiation into osteoclasts [11].

MDSCs promote prostate cancer bone metastases through immune suppression and differentiation into osteoclasts, fostering systemic accumulation and skeletal complications in tumor models [11]. Therapeutic targeting of MDSCs is effective, particularly when combined with sequenced therapies. In murine prostate models, Muralidhar et al. showed that androgen deprivation therapy (ADT) followed by 90Y-NM600 TRT outperformed the reverse order, as ADT-first preserved activated/memory CD4+ and CD8+ T cells while TRT-first drove MDSC accumulation and immune compromise [129]. Blocking CXCR2-mediated MDSC trafficking with sequencing further boosted survival. This approach, which limits MDSC recruitment, disrupts hypoxia-HIF-1α-NO-osteoclastogenesis, and combines immune-preserving sequences with MDSC inhibitors, preserved bone integrity in prostate (and breast/lung) cancer mouse models [129].

### 8.2. Clinical Models

Bone metastasis is a major feature of advanced cancer across many types of cancer, such as breast cancer, lung cancer, prostate cancer, etc. The molecular mechanisms of cancer spread to bone in human clinical models are similar to those in murine models [130]. Of the two major types of MDSCs, M-MDSCs are most responsible for bone metastasis because once they interact with cancer cells, they differentiate into immunosuppressive TAMs or metastasis-associated macrophages (MAMs) [103]. A study by Lasser et al. established that high MDSC levels in cancer patients correlate with weakened responses to immunotherapy [8]. As mentioned previously, human MDSCs express CD11b and CD33, markers associated with immunosuppressive functions [8].

Notably, however, the influence of MDSCs on immunity often differs subtly from one cancer to another. In breast cancer, for example, signals from metastatic breast cancer cells, such as RANTES and MCP-1, mediate the differentiation of MDSCs into functional, bone-resorbing osteoclasts [119]. In prostate cancer, the mediation of this process is mainly done by factors like HIF-1α and NO [11]. Despite these differences, the outcome is largely similar in different types of cancer, where the patient ends up experiencing osteolysis or bone resorption. When MDSCs differentiate into osteoclasts, they promote bone loss and destruction, thereby leading to skeletal-related events (SREs), including increased fracture risk and pain [131]. When this is combined with the immunosuppressive effect of MDSC that inhibits anti-tumor activity, it becomes easy for cancer to spread to the bones [11,131].

In the study by Bergenfelz et al., high levels of systemic M-MDSCs significantly correlated with de novo metastatic breast cancer and with liver and bone metastases. In this cohort, bone metastases were the most prevalent site, occurring in 78% of patients [132]. Notably, more patients with high M-MDSC levels had bone metastases than those with normal M-MDSC levels (92% vs. 64%, respectively) [132]. In another clinical study involving 126 patients with various cancers, the researchers found that patients with distant metastasis, including bone metastasis, had significantly higher levels of peripheral blood M-MDSCs than those with only lymph node metastasis [131]. Condamine et al. reviewed wide clinical data regarding metastasis and M-MDSCs across different cancers. The data revealed that circulating CD14^+^HLA-DR^lo^ M-MDSCs correlated with extra-thoracic metastases [102]. The general trend was that higher MDSCs were associated with more severe metastatic disease [102].

Elsewhere, in the study by Bergenfelz et al., the researchers also found a significant association between M-MDSCs and ER-negative tumors, with higher circulating Mo-MDSC levels associated with a greater likelihood of ER-negative tumors [132]. Among breast cancer patients with high levels of M-MDSCs, 41% were identified with ER-negative tumors, a proportion that was only 12% among patients with normal levels of M-MDSCs [132]. Similarly, more liver metastases were observed in 42% of patients with high M-MDSC levels compared with 18% in those with normal levels [132]. In a meta-analysis of large clinical datasets comprising 40 studies, MDSCs were significantly associated with worse OS (HR = 1.796, 95% CI = 1.587–2.032) [133]. In cases without pretreatment of circulating MDSCs, both prognosis and therapy were less effective [133]. The accumulation of MDSCs is a major factor not only in the progression of various cancers but also in their metastasis to other tissues, especially the bone [133]. Representative preclinical and clinical studies supporting MDSC accumulation and functional involvement in bone metastasis across tumor types are summarized in Table 1.

## 9. Tumor- and Lesion-Specific Nuances

MDSC biology in bone metastasis is tumor- and lesion-dependent. Most mechanistic data come from osteolytic breast cancer models, where osteoclast activity, TGF-β release, and the PTHrP–RANKL axis shape the niche. These findings may not fully extend to osteoblastic or mixed lesions, particularly in prostate cancer, where osteoblast-rich remodeling and distinct stromal cues likely influence myeloid populations differently. Thus, we use breast cancer as the best-studied example, not a universal model.

### 9.1. Breast Cancer

According to Huang et al., bone metastasis occurs in about 70% of advanced breast cancer cases, making it the most common site for distant dissemination of breast cancer [134]. These breast cancer bone metastases are, as noted above, predominantly osteolytic, driven by the PTHrP–RANKL–TGF-β axis. Rather than forming lesions, they cause bone resorption [135]. The initiator of osteoclast formation is PTHrP, secreted by breast cancer cells. Once PTHrP is secreted, it initiates or stimulates osteoblasts to express RANKL, which then leads to bone resorption by facilitating the differentiation and activation of osteoclasts [135,136]. Research also indicates that, by enhancing bone resorption, RANKL leads to the release of TGF-β and other growth factors from bones [136]. The TGF-β and other growth factors in the bone not only promote osteolysis but also enhance the dissemination and survival of tumor cells in the bone and upregulate PTHrP, leading to a vicious cycle that further damages the bones [136]. Therefore, the PTHrP–RANKL–TGF-β axis is central to breast cancer bone metastases. It explains why targeting RANKL has been the most reliable therapy for dealing with breast cancer bone metastases [137].

In breast cancer bone metastasis, there is extensive evidence of the immense role MDSCs play. According to Kim and Chakrabarti, the functionality of these cells is mainly through their immunosuppressive properties and their ability to compromise T-cell activity [138]. Additionally, MDSCs create a conducive environment for further tumor growth in the breast but also in other areas where the cancer cells are disseminated, including the bone. According to Kim and Chakrabarti, MDSCs are associated with increased CD11b^+^CD33^+^HLA-DR^lo^ in bone marrow adjacent to metastatic lesions, and they also secrete cytokines such as IL-6 and TNF-α, thereby facilitating osteoclast differentiation and activity [138]. In the study by Bergenfelz et al., 92% of breast cancer patients with high M-MDSCs (8.47–22.57%) had bone metastases, and only 64% of patients with low M-MDSCs had bone metastases [132]. Elsewhere, Sawant et al. established that MDSC depletion reduced osteolytic lesions by about 60% [119]. Given the wide research and wealth of available evidence linking MDSCs to breast cancer bone metastases, breast cancer is the best studied example for MDSC-driven bone disease, as it provides a framework upon which to understand the mechanism for causing bone metastasis.

### 9.2. Prostate Cancer

Unlike in breast cancer, bone metastases in prostate cancer are mainly osteoblastic in nature, characterized by abnormal bone density. The formation and dissemination of osteoblasts in prostate cancer are usually enhanced by several growth factors, including, but not limited to, endothelin-1 (ET-1), bone morphogenetic proteins (BMPs), Wnt signaling molecules, and IGF-1 and TGF-β [139]. The growth factors not only stimulate osteoblast proliferation and recruitment but also bone formation/deposition and osteoblast differentiation. The growth factors also contribute to the vicious cycle of continuous interaction between tumor cells and bone, thereby sustaining bone destruction. This type of bone metastasis, unique to prostate cancer, makes the bones structurally weak and prone to fractures [140].

Prostate cancer bone metastases are also immunosuppressive, but the mechanism of osteoblastic lesions’ impact on immunity may exhibit differences from osteolytic lesions. While the immune milieu may not be as well-defined and phenotyped as in breast cancer, osteoblasts have been reported to secrete cytokines, such as IL-6 and CXCL12, which often compromise immune cell function [141]. Bone damage, and the resulting activity of osteoblasts, create a welcoming environment for tumor cells, enabling cancer to spread further within the bone [139,141].

However, although wide research has shown that MDSCs accumulate in advanced prostate cancer, the specific mechanism of MDSCs’ function in prostate cancer has not been as widely studied as is the case in breast cancer. The interaction between MDSCs and osteoblast-driven lesions remains poorly explored, leaving a major research gap. The existing evidence is mostly descriptive or correlational. Still, it lacks strong clinical associations that may be necessary to enhance an in-depth understanding of the nuances and intricacies of MDSCs in prostate cancer. For instance, it is unclear whether osteoblast-derived factors recruit or activate MDSCs. Further, there is no overwhelming evidence showing whether MDSCs in prostate cancer differentiate to osteoclasts in the same way as they do in breast cancer. Therefore, prostate cancer is not adequately explored, which probably leaves space for further immunotherapy advancement, especially given that osteoblastic lesions are resistant to standard immune checkpoint blockade [142]. These differences likely influence MDSC biology. In osteolytic lesions, MDSCs are commonly linked to osteoclastogenic conversion and resorption-driven release of matrix factors. In osteoblastic lesions, dominant cues may instead arise from osteoblast-rich remodeling, altered cytokine gradients, and abnormal bone formation. Because direct comparative studies are limited, mechanisms defined in breast cancer bone metastasis should be applied cautiously to prostate cancer.

### 9.3. Lung Cancer

Lung cancer is not as prone to bone metastases as breast cancer or prostate cancer. However, when bone metastases occur, they can be as devastating as they are in other cancers, leading to poor patient outcomes. According to Popper, distant metastases in lung cancer are more likely to occur in parts such as the brain, liver, and adrenal glands than in bones [143]. Still, the frequency of their occurrence is significant, as Roato notes that in advanced non-small cell lung cancer (NSCLC), bone metastases occur in about 30 to 40% of lung cancer patients [144]. Given the relatively low frequency of bone metastases in lung cancer, their occurrence suggests a highly aggressive and/or advanced lung cancer.

Due to the relatively low occurrence of bone metastasis in lung cancer, there has been little research assessing its mechanism. However, existing research indicates that bone metastasis in lung cancer is highly associated with systemic immunosuppression [144]. In particular, MDSCs have been associated with immunosuppression in lung cancer, which leads to a higher likelihood of bone metastasis [144]. According to Xue et al., NSCLC patients with bone metastasis had higher levels of M-MDSCs and PMN-MDSCs than those without bone metastasis [145]. Like in breast cancer, MDSCs create immunosuppression, compromise T-cell activity, and enhance tumor growth. The local and systemic immune suppression enhanced by MDSCs in the bone microenvironment leads to bone damage, weakening the bone as the cancer advances.

Bone metastasis in lung cancer has a strong link to ICI resistance, a feature that is not necessarily as pronounced in other cancers. When treated with PD-1/PD-L1 inhibitors, NCSLC patients with bone metastases show poor response rates and low overall survival [146]. When the MDSC levels are high in lung patients with bone metastasis, the likelihood of poor ICI response increases significantly [146]. This can be explained by the aforementioned ability of the MDSCs to suppress anti-tumor immunity through the inhibition of CD8^+^ T-cell proliferation and the alteration of T-cell function. Additionally, MDSCs in NCSLCs increase ARG1 and iNOS expression and ROS activity, thereby suppressing anti-tumor immunity [146]. This further explains why patients with bone metastases show shorter PFS and overall survival when treated with ICIs.

### 9.4. RCC and Others

Single-cell RNA-sequencing (scRNA-seq) analyses of RCC primary tumors and metastatic sites, including the bones, have shown that MDSCs also play a crucial role in bone metastasis in RCC [147]. In RCC, MDSCs are characterized by high expression of *S100A8/A9*, *ARG1*, and *CXCR2* [148,149]. The MDSCs differentiate into TAMs and, as they acquire macrophage-like features, upregulate *CSF1R*, *MMP9*, and *IL1β* [147,150]. The MDSCs in RCC reinforce tumor survival in bone as they differentiate into TAMs [148]. The differentiation into TAMs is supported and enhanced by the marrow microenvironment, creating a vicious cycle that leads to bone destruction [147,151]. Therefore, although fewer data are available on bone metastases in RCC, scRNA-seq profiles indicate that MDSCs play a prominent role in these metastases.

In other cancers, MDSCs have also been found to be relevant in the formation of bone metastases. For instance, in melanoma, bone metastases are less common, but when they occur, they are associated with the presence of early myeloid progenitor populations transcriptionally similar to MDSCs [152]. As in other cancers where MDSCs have been studied, these populations differentiate into TAMs, compromising immunity and creating an environment favorable to metastasis [152]. Therefore, in RCC and other cancers, analyses of scRNA-seq data reveal that MDSCs are critical components in the formation of bone metastases [147]. While there may be tumor-specific skewing in each cancer, and the intricacies of tumor formation may differ slightly, the available data suggests that, across all cancers, MDSCs diversify into specialized suppressive myeloid subsets. However, a research gap exists because, other than in breast cancer, the functional cues from the bone microenvironment remain underexplored. Addressing these limitations will require emerging technologies and integrative analytical approaches. The following sections therefore highlight future directions for a better understanding of MDSC dynamics in bone metastasis.

## 10. Emerging Technologies and Future Directions

### 10.1. Single-Cell and Spatial Multi-Omics

The rise of single-cell analysis and high-throughput sequencing technologies offers promising avenues for a deeper understanding of MDSC heterogeneity [78]. Several studies have applied scRNA-seq to bone marrow metastasis across various primary tumor types, revealing broader stromal and immune interactions but generally lacking specific characterization of MDSC populations. For example, a study in RCC reported immune remodeling and enrichment of MDSCs but provided limited resolution of MDSC subsets and functions [147,153,154,155]. In addition, systemic factors can modulate these processes in a stage-dependent manner; G-CSF suppresses cancer cell homing to bone when administered before tumor seeding but accelerates metastatic progression after colonization, at least partly through MDSC-mediated mechanisms [80].

Single-cell profiling of pan-cancer bone metastases has already shown that the bone metastatic niche contains distinct myeloid and osteoclast-related states, including macrophage–osteoclast trajectories that support osteolytic remodeling. The same atlas also identified SELE-positive endothelial cells and exhausted CD8 T-cell states enriched in bone metastases, suggesting that myeloid suppression develops within broader spatially organized immune niches rather than as an isolated cell-intrinsic phenomenon [156]. In renal cell carcinoma bone metastases, scRNA-seq studies described MDSC-like cells with high *S100A8/A9*, *ARG1*, and *CXCR2* expression that could shift toward TAM-like phenotypes with *CSF1R*, *MMP9*, and *IL1β* expression, supporting the idea that suppressive myeloid cells in bone can adopt progressively more macrophage-like states [101]. Single-cell analysis of metastatic bone marrow in neuroblastoma also revealed enrichment of tumor-associated neutrophils, macrophages, exhausted T cells, and Tregs, along with reduced B cells, illustrating how bone metastatic niches can become broadly immunosuppressive and myeloid-skewed [154]. In parallel, spatial multi-omics technologies preserve tissue spatial organization, enabling cells to be studied within their native microenvironment [157]. This is particularly valuable for MDSC research, as it facilitates identification of MDSC niches and cellular neighborhoods, allowing improved mapping of MDSC subsets, better discrimination between PMN-MDSCs and M-MDSCs, and trajectory inference through lineage tracing and barcoding approaches [154,158]. Multimodal spatial profiling of metastatic breast cancer biopsies has already shown that macrophage populations and T-cell infiltration/exclusion states are spatially organized within lesions, demonstrating how spatial methods can reveal suppressive immune neighborhoods that are difficult to appreciate from dissociated single-cell data alone [157].

The study of bone metastases will further expand our understanding of these skeletal complications and the cellular mechanisms underlying them [155]. This progress is largely driven by single-cell multi-omics approaches that integrate multiple layers of molecular information from individual cells, enabling more comprehensive characterization of cellular identity and function [155]. Techniques such as scRNA-seq, single-cell ATAC-seq, CITE-seq, and REAP-seq provide insights into gene expression patterns, transcriptional states associated with MDSC functionality, and differentiation trajectories. In parallel, spatial multi-omics technologies preserve the spatial organization of tissues, enabling cells to be studied within their native microenvironment [157]. This is particularly valuable for MDSC research, as it facilitates identification of MDSC niches and cellular neighborhoods, allowing improved mapping of MDSC subsets, better discrimination between PMN-MDSCs and M-MDSCs, and trajectory inference through lineage tracing and barcoding approaches [157].

### 10.2. Functional Imaging and In Vivo Tracking and Whole-Body MRI with Diffusion-Weighted Imaging

Functional imaging and in vivo tracking technologies have also proven highly useful for studying bone metastases, particularly regarding the role of MDSCs [158]. Through these technologies, it is possible to track the trafficking of MDSCs from bone marrow to metastatic bone lesions and other distant organs [158]. Consequently, these technologies enable researchers to gain an in-depth understanding of how MDSCs orchestrate immunosuppression and metastatic progression. An example of the application of functional imaging has been genetic and fluorescent lineage tracing, which has been used in preclinical models. This has been done by genetically marking MDSC-like populations, then tracking them during metastatic progression [158].

Another technique that has continued to advance and facilitate the study of bone metastasis is whole-body MRI with diffusion-weighted imaging, which enables non-invasive imaging and tracking of changes in cellular density within bone marrow and bone lesions [159]. Multimodal imaging approaches, such as optical imaging and multimodal probes, combine several techniques to enhance MDSC tracking. Techniques such as multimodal probes, nanoparticle-labeled cell labeling, reporter gene systems, and chemokine receptor imaging enhance in vivo tracking of MDSCs [160]. These strategies facilitate different approaches to assessing and tracking MDSCs, including studying their pathways, tracking MDSCs themselves, and real-time visualization of their migration from marrow to bone lesions [160].

### 10.3. Biomarkers

Analysis of MDSC subsets, particularly M-MDSCs and PMN-MDSCs, in peripheral blood has emerged as a valuable liquid biopsy approach for monitoring bone metastases [131]. These cells reflect systemic tumor-host interactions and offer a minimally invasive means of assessing disease burden [150]. Consistent with the mechanisms outlined above, circulating MDSC levels correlate directly with the extent of bone involvement, with higher counts indicating greater metastatic spread [161].

Measurement typically involves determining the absolute counts or frequencies of M-MDSCs and PMN-MDSCs in blood. Building on the mechanisms discussed earlier, these same parameters can also help identify impending SREs, as elevated MDSC levels are linked to OCs activation and increased bone resorption [132]. Functional markers in MDSCs, including ARG1, ROS production, and RANKL, contribute to immunosuppressive and osteoclastogenic effects that promote bone resorption in cancer and musculoskeletal disorders, potentially exacerbating skeletal complications such as fractures and spinal cord compression [60]. Integrating MDSC functional markers with bone turnover markers (e.g., CTX for resorption, PINP for formation) could provide complementary insights into bone remodeling and metastatic progression [11]. However, their combined prognostic value for skeletal complications has not been validated.

Given MDSC’s potent immunosuppressive activity, circulating MDSC levels have also been investigated as potential indicators of ICI response and resistance. Baseline and on-treatment levels of circulating MDSC subsets are measured. In doing so, high circulating MDSC levels are consistently associated with a poor response to ICIs, as MDSCs are potent inhibitors of T-cell activation and trafficking [146]. Consequently, MDSCs exert an immunosuppressive effect, thereby reducing the efficacy of the ICI. Additionally, the expression of suppressive mediators, including ARG1, PD-L1, and IL-10–related signatures, is measured when evaluating ICI response and resistance [104] (Figure 3D).

### 10.4. Integrative Systems-Biology Models

More recently, efforts have been made to develop integrative systems-biology models that further enhance the ability to mark metastatic cancer and related elements, including MDSCs. These advanced technologies can capture aspects that earlier technologies could not, such as the multi-scale interplay between bone remodeling, myelopoiesis, and systemic immunity in the context of bone metastases [162]. Therefore, these technologies introduce a new approach to predictive modeling by integrating cellular, tissue, and organism-level data into a single predictive framework. These models are mainly based on mathematical or computational calculations that employ ordinary differential equations (ODEs) or agent-based approaches to explore the complex network of feedback loops that collectively define bone remodeling [163]. These mathematical models maintain balance between bone formation and resorption by simulating the interactions among osteoclasts, osteoblasts, and osteocytes [164]. By creating mathematical models that express the creation of myeloid-biased hematopoiesis, it becomes possible to investigate how changes in bone integrity propagate upward to influence immune cell production.

These integrative models have been deployed at the cellular level to study the behavior and fate of specific cells within the bone marrow in metastatic cancer. For instance, the process of myelopoiesis, during which HSCs differentiate into mature myeloid cells, is tracked using integrative models to assess potential bias toward suppressive lineages [165]. Differential equations that define progenitor proliferation, differentiation, and egress into circulation are used to track MDSC expansion, enabling these models to predict MDSC levels and their impact on metastatic burden. The integrative systems-biology models can also mimic MDSC trafficking and illustrate their immunosuppressive effects, providing a basis for investigating and may help explain the link between observed MDSCs and bone metastases. Further, by using multi-scale models, investigators can accurately capture how circulating MDSCs dampen T-cell and NK-cell activity [166].

One major advantage of integrative systems-biology models is that they can integrate diverse data streams, something that other strategies cannot [167]. These models can also, on their own, turn descriptive data into predictive insight, making them useful for a wider variety of purposes and with increased precision. Additionally, these models can be developed for patient-specific situations to simulate how the disease would respond to various interventions, thereby creating a basis for choosing the most suitable intervention [166].

## 11. Future Directions

### 11.1. Bone-Specific Transcriptional Programs in MDSCs as Therapeutic Targets

Recent evidence demonstrates that bone-specific transcriptional programs in MDSCs can potentially be targeted therapeutically. MDSCs in the bone microenvironment function by adopting bone-specific transcriptional programs driven by signals from osteolytic and bone marrow environments [44]. These programs that shape MDSCs are either pre-programmed to endow MDSCs with innate suppressive characteristics or induced in bone metastatic niches. Therefore, when developing interventions targeting these programs, it is important to use a bone-specific approach.

### 11.2. Bone Marrow Niche Normalization to Reverse Systemic Immunosuppression

Normalization of the bone marrow niche, which involves restoring balanced hematopoiesis, can indeed reverse systemic immunosuppression. Normalization would restore the proper balance between osteoclasts and osteoblasts and minimize inflammatory and tumor-derived signals [44]. Additionally, normalization would restore immune-supportive stromal interactions [44,168]. These actions would disrupt the vicious cycle that causes erroneous myelopoiesis, MDSC expansion, and immune dysfunction. However, the extent to which absolute normalization can be achieved is debatable. Moreover, normalization alone may not be sufficient because of tumor-intrinsic mechanisms and established immunosuppressive networks outside the marrow that cannot be addressed using normalization [44].

### 11.3. Optimal Sequencing of MDSC-Targeting Combination Therapies

The most plausible combination therapy for MDSC-targeting involves deploying agents that deplete or reprogram MDSCs, such as ICIs, in combination with standard bone-protective therapies [168]. This approach would help reduce MDSC generation and potentially deplete them, while normalizing the bone marrow niche to avoid rebound myelopoiesis and to overcome the immunosuppressive vicious cycle that destroys the bone environment.

## 12. Conclusions and Clinical Implications

Bone metastasis should be viewed not only as a secondary tumor site, but as a marrow-centered “MDSC amplifier” that actively reshapes hematopoiesis and the immune landscape in ways that promote skeletal colonization, immune escape, and treatment failure. Across tumor types, metastatic cues remodel key marrow niches, sinusoidal/vascular interfaces, CXCL12-rich stromal and osteolineage compartments, and adipocyte-driven metabolic environments, thereby recruiting myeloid precursors, stabilizing suppressive programming (e.g., ARG1/ROS/NO and checkpoint ligand expression), and in some contexts coupling immunosuppression to osteoclastogenic activity that accelerates bone destruction. Clinically, this framework helps explain why bone-dominant disease is frequently associated with a “cold” immune microenvironment and diminished responsiveness to immune checkpoint blockade, because the marrow is uniquely positioned to export bone-conditioned suppressive myeloid populations systemically while simultaneously reinforcing local T-cell dysfunction, Treg skewing, and impaired innate cytotoxicity. The translational implication is that effective immunotherapy for patients with bone metastases will likely require rational combinations that (i) limit MDSC trafficking and retention, (ii) deplete or reprogram suppressive myeloid states without prohibitive marrow toxicity, and (iii) disrupt bone-resorptive feedback loops using bone-directed agents, with attention to sequencing and biomarkers that capture MDSC states rather than bulk myeloid counts. Finally, emerging single-cell, spatial, and integrative modeling approaches provide a realistic path to stratify patients, map bone-specific MDSC heterogeneity, and identify actionable nodes for testing in mechanism-driven clinical trials aimed at restoring antitumor immunity while reducing skeletal-related events.

## Figures and Tables

**Table 1:** Summary of evidence for MDSC accumulation and function in bone metastases.

Tumor Type	Model	Evidence Level	MDSC Subset	Main Findings in Bone Metastases	Functional Implications
BREAST CANCER [120]	4T1 (fLuc) BALB/c mouse model	Preclinical	CD11b+Gr-1+ MDSCs	MDSCs accumulated in metastatic bone and adjacent marrow; bone marrow MDSCs differentiated into osteoclasts.	Bone niche promotes osteoclastogenesis and bone destruction.
BREAST CANCER [13]	PTH1R/PTHrP murine model	Preclinical	M-MDSCs	Tumor-derived PTHrP mobilized M-MDSCs from bone marrow; PTH1R activation disrupted osteoblast–M-MDSC adhesion via ADAM17/MMP7.	Bone stromal signaling controls M-MDSC release and metastatic support.
PROSTATE CANCER [11]	Murine bone metastasis models	Preclinical	MDSCs, mainly M-MDSCs	MDSCs accumulated in bone lesions and promoted osteoclast differentiation; HIF-1α/NO amplified this process.	Immune suppression and osteoclastogenesis drive skeletal disease.
PROSTATE CANCER [130]	ADT followed by 90Y-NM600 TRT model	Preclinical	MDSCs	ADT-first reduced MDSC-driven immune compromise; TRT-first increased MDSC accumulation. CXCR2 blockade improved survival.	MDSC targeting preserves immunity and bone integrity.
BREAST CANCER [133]	Clinical cohort	Clinical	M-MDSCs/Mo-MDSCs	High systemic M-MDSCs associated with metastatic breast cancer and bone metastasis; bone metastases were more frequent with high M-MDSCs.	Circulating M-MDSCs may indicate metastatic burden.
MIXED CANCERS [132]	Clinical study	Clinical	Peripheral blood M-MDSCs	Patients with distant metastasis, including bone involvement, showed higher M-MDSC levels than those with nodal disease only.	Higher M-MDSCs track with advanced metastatic spread.
MIXED CANCERS [106,134]	Clinical review/meta-analysis	Clinical	CD14+HLA-DRlo M-MDSCs	Circulating M-MDSCs correlated with extra-thoracic metastases; higher MDSCs linked to worse overall survival.	MDSCs are associated with poor prognosis and reduced treatment response.

## Data Availability

No new data were created or analyzed in this study. Data sharing is not applicable to this article.
